# Editorial: Design, Synthesis, and Preclinical Testing of Innovative Anti-Cancer Compounds With a High Level of Selectivity of Action and Low Toxicity

**DOI:** 10.3389/fmolb.2022.859821

**Published:** 2022-04-07

**Authors:** Ana Maria Da Costa Ferreira, Regine Schneider-Stock, Francesca Aiello

**Affiliations:** ^1^ Department of Chemistry, University of São Paulo, São Paulo, Brazil; ^2^ Institute of Pathologie, Erlanger University, Erlanger, Germany; ^3^ Department of Pharmacy, University of Calabria, Cosenza, Italy

**Keywords:** anti-cancer (anticancer) drugs, innovative compounds, strategies to improve biological activity, metabolomics, selectivity

The searching for novel anti-cancer compounds has deserved much interest in the literature, and many efforts from different groups around the world were spent in the last decades to develop them. Diverse used approaches focused both small organic molecules as well as corresponding metallated ligands, at their reactivity toward a range of biological targets. In this Research Topic of Frontiers in Molecular Biosciences, under the title *Design, Synthesis, and Preclinical Testing of Innovative Anti-Cancer Compounds* we intended to motivate publications in innovative anticancer compounds, aiming to reach high level of selectivity and low toxicity. Despite of only a few articles reached these goals, they are representative of recent improvements in the field. Such studies utilized advanced methodology, in accordance with recommended recent procedures in the literature. Three of the published works focus on metal compounds of ruthenium, copper and/or platinum. Metalation usually improve the activity of ligands, providing charge and facilitating their uptake by the cells. Also, the binding of metal ions to those ligands favored an specific conformation, frequently assisting its interaction with main targets as DNA or the active sites of proteins co-involved in crucial processes. On the other hand, coordinated ligands modify the metal reduction potential, by modification of the reduced/oxidized species ratio, and so influencing the oxidative processes that usually occurs more intensely in tumor cells. The continuous progress of studies based on metal complexes in the last decades, included some compounds tested in clinical trials.

In The Influence of Some Axial Ligands on Ruthenium–Phthalocyanine Complexes: Chemical, Photochemical, and Photobiological Properties (Martins et al.), the authors prepared and described chemical and photochemical properties of ruthenium-phthalocyanine complexes, particularly *trans*-isomers. Ruthenium is one of the most studied metals in the development of metallodrugs for cancer, particularly due its photochemical reactivity and kinetic characteristics that can differentiate its modes of action. Usually, the possible substitution of ligands in biological medium interferes considerably in the anticancer activity of ruthenium complexes and this fact is more important than the reduction of the metal. Usually, the possible substitution of ligands in biological medium interferes considerably in the anticancer activity of ruthenium complexes. And this point is more important than the reduction of the metal. Particularly, results for one of the compounds studied indicated triplet excited-state lifetimes, measured by nanosecond transient absorption, occurring as two processes, one around 15 ns, and the other around 3.8 μs. Therefore, some modulation of the metal complex properties can lead to better potential therapeutics.

In the review Anticancer Compounds Based on Isatin-Derivatives: Strategies to Ameliorate Selectivity and Efficiency (Ferraz de Paiva et al.), the authors described and compared the wide biological properties of an ubiquitous natural compound isatin. Isatin itself and many derivatives exhibit a range of biological properties, acting mainly as antitumor agents. Different strategies to improve significantly the antiproliferative activity of these derivatives were presented, including functionalization at different positions of the oxindole ring, reactive oxygen species (ROS) generation, and metalation by diverse ions. Compounds of this type that already entered in clinical tests and influenced many further studies were displayed, and its undesirable side effects pointed out, motivating innovations. The studies also discussed possible mechanisms of action of such compounds, as DNA binding and damage, mitochondria interactions, and inhibition of selected proteins (as kinases and topoisomerases). Discussions about the influence of the metal (copper and zinc) and of ligand features in the reactivity of these compounds indicated a modulation of their anticancer activity. Further, the insertion of such metallated and non-metallated compounds in suitable matrices was focused, displaying how the matrices can act as significant adjuvant agents, protecting and/or modifying their activities toward tumor cells.

Complementing those studies, a metabolomic investigation in the anticancer effects of cisplatin and of copper compounds known as Casiopeinas appeared in ^1^H -NMR Metabolomics Study of the Effect of Cisplatin and Casiopeina IIgly on MDA-MB-231 Breast Tumor Cells (Resendiz-Acevedo et al.). Metabolomics, placed downstream of genomics, transcriptomics, and proteomics, improves the system-level view by metabolites assay, allowing the determination of their concentration, and metabolic pathways. The metabolomics field continues to develop rapidly, constituting an important tool in scientific research for structural, qualitative, and quantitative analyses of bio-macromolecules, such as proteins and nucleic acids, as well as small molecules, or phytochemicals. By now, the NMR-based approaches are becoming indispensable for the identification of known and unknown metabolites, cell and tissue extracts, in whole organisms. Both complexes focused in this study were compared in relation to its reactivity toward breast tumor cells. The metabolic profile of hormonal-independent triple-negative breast cancer cells (MDAMB-231) was followed, using 1H-NMR spectroscopy, after treatment for 20 min with cisplatin, or 40 min with Casiopeina IIgly, in contrast to non-treated cells. Both complexes showed different modes of action, with a preferential binding to DNA in the case of platinum, or adding oxidative damage ability in the case of copper. The results indicated that both compounds had different effects over the metabolism of the tumor cells. Particularly, the copper compounds seem to act preferentially in the carbohydrate and nucleotide metabolisms. Inter-correlations between metabolic routes and typical cancer cells pathways, as apoptosis, metastasis, and migration, were also provided.

Finally, the cytotoxicity of CAR-T-cells was focused in the work IL-21 Optimizes the CAR-T Cell Preparation Through Improving Lentivirus Mediated Transfection Efficiency of T Cells and Enhancing CAR-T Cell Cytotoxic Activities (Du et al.). Individualized cancer immunotherapy is a trend more recently in oncology, and CAR-T-cell therapy constitute promising advanced methodology in this direction. Therefore, optimize the production methods to obtain CAR-T cells with advanced quality are crucial for antitumor immunotherapy research. In this pioneer study, it is discussed how the addition of IL-21, commonly used in cytokine cocktails, can enhance lentiviral transfection efficiency of T cells. Consequently, an additional optimized preparation method was developed, attesting the benefits of IL-21 in the CAR-T cell preparation prior to their use in adoptive T-cell therapy. The design of future clinical trials can probably apply results from this study.

Perspectives of further investigations in anticancer compounds are still promising. There are huge possibilities of using diverse natural or synthetic molecules, besides corresponding complexes metallated with different metal ions that usually increase their anticancer properties. The herein focused articles deserved up to 11,800 total views, and 15 citations until March 2022. We hope that these examples of studies here discussed can encourage the syntheses of innovative compounds, and promote a continuous progress of the field.

